# Interprofessional collaboration: Measuring occupational therapists and teachers’ perceptions of collaborative practice in inclusive Australian primary schools

**DOI:** 10.1177/03080226241295601

**Published:** 2025-02-23

**Authors:** Jill Jeremy, Joanne Hinitt, Ilektra Spandagou

**Affiliations:** 1 Sydney School of Education and Social Work, Faculty of Arts & Social Sciences, The University of Sydney, Camperdown, Australia; 2 Sydney School of Health Sciences, Faculty of Medicine & Health, The University of Sydney, Sydney, Australia; 3 Sydney School of Education & Social Work, Faculty of Arts & Social Sciences, The University of Sydney, Sydney, Australia

**Keywords:** Interprofessional collaboration, occupational therapists, teachers, inclusive education, evaluation, measurement

## Abstract

**Introduction:**

Globally occupational therapists are collaborating with teachers to support the inclusion of students with disabilities in mainstream schools. To begin to understand how collaboration promotes inclusion, this study aimed to measure occupational therapists and teachers perceived collaborative practice.

**Method:**

A quantitative cross-sectional analysis of occupational therapists and teachers in mainstream primary schools in three Australian states was conducted via an anonymous online survey. Eligible participants were recruited via self-selection and snowballing, resulting in a nonprobability sample of 108 occupational therapists and 33 primary teachers. The Teacher – Therapist Collaboration Index, an instrument based on an existing conceptual framework and associated tool was developed to measure perceived collaborative practice.

**Results:**

Occupational therapist and teacher profiles were similar. Both professions report above average collaborative practice, although teachers rated themselves more collaborative on two components. Ratings did not significantly differ by profession, demographic, or background. Personal, professional and systems influences positively correlated with collaboration ratings, with systems having the strongest relationship.

**Conclusion:**

Systems changes may be necessary to improve collaboration. Therapists could use the framework and measurement instrument as tools to plan, execute and evaluate their collaborative practice.

## Introduction

Improving school inclusion requires education systems to transform their culture, policies and practices to eliminate barriers to learning (United Nations, 2006, 2016). As occupational therapists are crucial to this inclusive education transformation (World Federation of Occupational Therapists (WFOT), 2016), school-based occupational therapy has grown (American Occupational Therapy Association, 2020) as has the expectation that therapists work collaboratively with teachers within the classroom context (Jeremy et al., 2024a). Evaluating the effectiveness of interprofessional collaboration in fostering inclusion requires accurate measurement of the practice. This necessitates valid and reliable measurement instruments grounded in a conceptual framework and validated within the context of schools and with both teachers and occupational therapists (Ianni et al., 2022; Jacob et al., 2017; Mellin et al., 2010). Conceptual frameworks provide a theoretical foundation for defining and operationalising collaboration constructs, ensuring that measurement tools accurately capture the intended concepts. Validation with both teachers and occupational therapists within the school context ensures that instruments are relevant and applicable to both professions working in inclusive education settings.

The lack of psychometrically sound instruments grounded in a conceptual framework and validated with multi-disciplinary stakeholders within the school context is a known challenge to measuring interprofessional collaboration in education settings (Ianni et al., 2022). Although several studies have attempted to measure collaboration between teachers and occupational therapists (Barnes and Turner, 2001; Edick et al., 2022; Friedman et al., 2022; Huang et al., 2011; Kennedy and Stewart, 2012; Rens and Joosten, 2014), only one (Friedman et al., 2022) employed a validated measurement tool based on a conceptual framework of collaboration. The remaining studies used atheoretical study-specific measurement tools (Jeremy et al., 2024a). To advance our understanding of interprofessional collaboration, there is a critical need for studies that utilise validated measurement tools rooted in a conceptual framework.

### Study aim

This study aimed to measure perceived collaborative practice between teachers and occupational therapists using a measurement tool grounded in a conceptual framework and validated within the school context by both professions. The study sought to rate perceived collaborative practice, compare the collaboration ratings between professions, and examine the relationship between collaboration ratings and different variables, including personal, professional and systems factors.

## Method

Ethical approval was obtained from the University of Sydney Human Research Ethics Committee (Project Number 2021/278) prior to the commencement of recruitment and survey distribution. Written informed consent was obtained from study respondents prior to participation.

### Design and instruments

The study used a quantitative cross-sectional design using an anonymous online survey. The entire survey was piloted by five experienced occupational therapists and five teachers whose feedback helped improve the structure, layout and relevance of the questions. The refined survey questionnaire comprised 70 questions over four sections:

- Section 1: Demographics and background data
- Section 2: Teacher-Therapist Collaboration Index
- Section 3: Influences on collaboration
- Section 4: Additional comments

### Selecting a conceptual framework and measurement instrument

A scoping review (author, date) identified six studies that used an instrument to measure occupational therapist – teacher collaboration in school settings. Five studies (Barnes and Turner, 2001; Edick et al., 2022; Huang et al., 2011; Kennedy and Stewart, 2012; Rens and Joosten, 2014) used tools that did not have a theoretical foundation and were thus deemed unsuitable. Although the remaining study (Friedman et al., 2022) used an instrument based on a conceptual framework, it was also considered unsuitable as it was validated for interprofessional education initiatives between healthcare professions in healthcare settings. Consequently, the review was expanded to include measurement tools used by professionals beyond occupational therapists and teachers.

Two systematic reviews of collaboration models and measurement tools were located. Hillier et al.’s (2010) review identified 34 studies reporting or evaluating models for successful interactions between educators and health professionals. The review revealed a continuum of interaction models, ranging from consultative to interactive teaming. It noted that models were not well evaluated. No models were based on a conceptual framework of collaboration. Jacob et al.’s (2017) review identified and evaluated the psychometric properties of 10 tools that measured interprofessional collaboration between professionals from health and other disciplines. Two predominant interdisciplinary conceptual frameworks, and corresponding psychometrically sound measurement instruments, were identified (Bronstein, 2002, 2003; Ødegård, 2005, 2006). As no conceptual framework describes all components of the phenomenon of collaboration (Ødegård, 2005), either framework would have been suitable for the study; however, Bronstein’s (2003) model was selected due to its simplicity, multi-disciplinary perspectives, theoertical underpinnings and unit of analysis.

**Figure 1.**
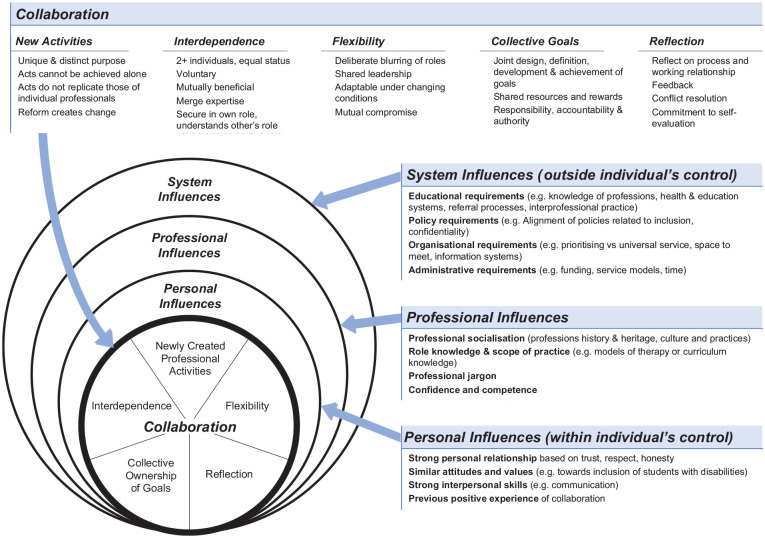
A framework of interprofessional collaboration and the influences on it. Source: Adapted from Bronstein (2003).

Bronstein’s (2003) Model for Interdisciplinary Collaboration, conceptualised in the field of social work, is a generic depiction of components of ideal collaboration amongst professionals from different disciplines. The model is based on four separate theoretical frameworks and describes five components of collaboration: interdependence, newly created professional activities, flexibility, collective ownership of goals and reflection on process, as well as four influences on collaboration: professional role, structural characteristics, personal characteristics and a history of collaboration. Although Bronstein’s (2003) model included four factors that influence collaboration, a review of the literature (Jeremy et al., 2024a) identified further influencing factors, thus, the framework was modified to include three broad categories of influence: personal, professional and systems influences. Figure 1 illustrates the conceptual framework employed for the study.

## Development of the Teacher-Therapist Collaboration Index

Bronstein’s (2003) conceptual framework has been operationalised through various measurement instruments which use item statements to measure the five components of collaboration. The existing instruments, the Index of Interdisciplinary Collaboration (Bronstein, 2002), the Index of Interprofessional Team Collaboration for Expanded School Mental Health (IITC-ESMH; Mellin et al., 2010), and the Modified Index for Interdisciplinary Collaboration (MIIC) (Oliver et al., 2007), were reviewed and compared but were deemed unsuitable for the study due to reasons of length, complexity and wording. A new 30-item instrument, the Teacher-Therapist Collaboration Index (TTCI), was developed. Based on a scoping review (Jeremy et al., 2024a) and the existing instruments, the TTCI was created by carefully selecting and adapting items to ensure breadth, clarity and brevity. The wording of items in each instrument was compared to ensure they measured the same concepts. Tables 1 and 2 present a comparison of the TTCI with the existing instruments. Like the existing instruments, the TTCI items use a 5-point Likert scale to which respondents rate their agreement (1 = Strongly agree to 5 = Strongly disagree). Higher levels of collaboration are reflected by lower scores on the TTCI, with a score of 30 being most collaborative, 150 least collaborative and 90 the median.

**Table 1.** Comparison of collaboration instruments.

Instrument name, Author, Date	Total no. of items	Number of items in each component					Scale	Validated with Occupational Therapists and/or Teachers	Level (Team, Dyad, Individual)
		Interdependence	Newly created professional activity	Flexibility	Collective ownership of goals	Reflection on process			
Index of Interdisciplinary Collaboration (IIC), Bronstein (2002)	49	16	7	6	9	11	5-point Likert (1 = Strongly Agree, 5 = Strongly Disagree)	Neither	Individual (“I”)
Modified Index for Interdisciplinary Collaboration (MIIC), Oliver et al. (2007)	42	13	6	5	8	10	5-point Likert (1 = Strongly Agree, 5 = Strongly Disagree)	Neither	Individual (‘I’)
Index of Interprofessional Team Collaboration for Expanded School Mental Health (IITC-ESMH) Mellin et al., (2010)	26	9	4	4	1	8	5-point Likert (1 = Strongly Agree, 5 = Strongly Disagree)	Teachers only	Team (‘The team’)
Teacher-Therapist Collaboration Index (TTCI) Jeremy et al., (2024b)	30	9	4	5	5	7	5-point Likert (1 = Strongly Agree, 5 = Strongly Disagree)	Both	Dyad (“We”)

**Table 2.** Comparison of wording of items between instruments.

Component	IIC	MIIC	IITC-ESMH	TTCI
Interdependence	*I* can define those areas that are distinct in my professional role from that of professionals from other disciplines with whom I work	*I* can define those areas that are distinct in my professional role from that of professionals from other disciplines with whom I work	*The team* makes distinctions among the roles and responsibilities of each member	*We* make distinctions between us regarding our roles and responsibilities
Newly created professional activity	Distinct new programmes emerge from the collective work of colleagues from different disciplines	Working with colleagues from other disciplines leads to outcomes that we could not achieve alone	New practices related to working with youth occur as a result of the diversity of ideas among team members	New practices related to working with students occur as a result of the diversity of ideas between us
Flexibility	I am willing to take on tasks outside of my job description when that seems important	I am willing to take on tasks outside of my job description when that seems important	Team members take on tasks outside their role when necessary	We take on tasks outside our role when necessary
Collective ownership of goals	My interactions with colleagues from other disciplines occurs in a climate where there is freedom to be different and to disagree	My interactions with colleagues from other disciplines occurs in a climate where there is freedom to be different and to disagree	There is freedom to be different and disagree within the team	We are free to disagree with each other
Reflection on process	***My colleagues from other disciplines and I*** often discuss different strategies to improve our working relationships	***My colleagues from other disciplines and I*** often discuss different strategies to improve our working relationships	***Team members*** discuss strategies to improve their working relationship	***We*** discuss strategies to improve our working relationship

IIC: Index of Interdisciplinary Collaboration; MIIC: Modified Index for Interdisciplinary Collaboration; IITC-ESMH: Index of Interprofessional Team Collaboration for Expanded School Mental Health; TTCI: Teacher-Therapist Collaboration Index.

## Development of the influences on collaboration instrument

Interprofessional collaborative practice is influenced by contextual factors. Understanding the relationship between collaboration and personal, professional and system factors requires being able to measure these. No instrument to measure these factors was identified (author, date); therefore, one was developed in consultation with professionals experienced in collaborative practice. This 19-item instrument, influences on collaboration, included 8-items regarding personal influences, 8-items regarding professional influences and 3-items regarding system influences. Consistent with the TTCI, agreement is indicated through a five-point Likert scale (1 = Strongly agree and 5 = Strongly disagree). Positive influences on collaborative practice are reflected by lower scores on the index. A total score of between 19 and 57 indicates factors are acting as facilitators, while a total score greater than 57 indicates they are acting as barriers.

## Establishing validity and reliability scales

To ensure the validity and reliability of the instruments, a process of scale development and piloting was conducted. Content validity was established through expert consultation and literature review (Creswell, 2014). Criterion validity was assessed by correlating scores with established measures of similar constructs. While inter-rater reliability was not explicitly assessed, the pilot did not reveal any inconsistency between raters.

Internal consistency analysis was conducted using Cronbach’s alpha (DeVellis, 2003). This indicated moderate reliability for the TTCI (0.894) and its subscales (0.774–0.887). These results, comparable to the existing instruments, suggest the TTCI effectively captures aspects of collaboration and is a valid and reliable instrument for assessing interprofessional collaboration. The influences scale showed moderate internal consistency (Cronbach’s α = 0.866), with subscales demonstrating acceptable consistency, 0.698 for systems influences, 0.771 for personal influences and 0.828 for professional influences. As Cronbach α values are sensitive to the number of items in the scale it is common for short scales, such as these, to have low Cronbach α values.

### Sample

While probability sampling is preferred for ensuring generalisability, it requires a complete and current list of the target population (Sharma, 2017). Due to the unknown number of occupational therapists and teachers collaborating in Australian schools, obtaining such a list was impractical. Therefore, non-probability sampling methods, including convenience sampling, self-selection and passive snowballing, were used. Participants were recruited through social media, professional networks, advertising and targeted emails. As sample size was not predetermined, participants were sought until survey discontinuation. One hundred and eight occupational therapists and 33 teachers participated in the study. Study participants worked in primary schools and were qualified and registered to practise in their respective state.

### Data collection

A 17-month data collection period during COVID-19 ensured participation despite pandemic-related impediments. The anonymous online survey questionnaire was securely hosted by REDCap and accessed via a generic link. Participants provided electronic consent prior to completing the survey.

### Data analysis

Data were analysed using IBM SPSS Statistics (Version 28). Some participants did not answer every question; therefore, analysis was based on the number of responses received. Descriptive statistics, frequencies and percentages were used to describe the demographic and employment characteristics of respondents. Due to the non-probability sampling technique, and the small, unequal sample size and as data violated assumptions of normality (Pallant, 2020), non-parametric tests were selected as the most appropriate for analysing inferential statistics.

## Results

Results should be interpreted and generalised with caution due to reasons outlined in the limitations section.

### Demographics and background characteristics of participants

Table 3 presents the demographics and background data. A separate paper reports the profile of occupational therapist participants (Jeremy et al., 2024a). Most respondents, 59.4% of teachers and 54% of therapists, practised in the state of New South Wales. The profiles of both professions were similar. Most respondents were female, aged between 26 and 35 years, with a bachelor’s degree, and engaged in full-time practice. Experience differed between the two groups, with 46.7% teachers having between 10- and 19-years’ experience, while 46.7% of therapists had less than 10 years. Most teachers (75.9%) were government employees, while the majority of therapists (64%) worked in private practice. Models of therapy service varied between teachers and therapists. The most common service was direct 1:1 therapy or consultation for an individual student.

**Table 3.** Demographics and background characteristics.

Characteristics	Teachers		Occupational Therapists	
	*n*	%	*n*	%
State of employment				
NSW	19	59.4	57	54
QLD	9	28.1	18	17
VIC	4	12.5	30	29
Total*	33	100	105	100
Gender				
Male	2	6	6	6
Female	31	94	100	94
Total*	33	100	106	100
Age (years)				
Under 25	0	0	11	10.5
26–35	11	34.5	45	42.9
36–45	8	25	29	27.6
46–55	10	31	12	11.4
56–65	3	9.5	8	7.6
Total*	32	100	105	100
Highest level of education				
Diploma	1	3	1	1
Bachelor	24	73	75	71
Master	8	24	30	28
Total*	33	100	106	100
Employment hours				
Full-time (⩾38 hours/weeks)	24	73	58	55
Part-time (<38 hours/weeks)	9	27	48	45
Total*	33	100	106	100
Years in profession				
Less than 10	9	30	49	46.7
10–19	14	46.7	36	34.3
20–29	4	13.3	10	9.5
30–39	3	10	9	8.6
40–49	0	0	1	1
Total*	30	100	105	100
Employer (Occupational Therapist)				
Government Health			1	1
Government Education			14	16
Private practice			58	64
Agency/Not for profit			14	16
Other			3	3
Total*			90	100
Employer (Teacher)				
Government system	22	75.9		
Catholic system	6	20.7		
Independent system	1	3.4		
Total*	29	100		
Model of therapy service				
Direct 1:1	12	42	54	60
Direct small group	4	14.3	3	3.4
Indirect	5	17.9	4	4.5
Consultation for an individual	6	21.4	25	28.1
Consultation for a class	1	3.6	3	3.4
Consultation for a school	0	0	0	0
Total*	28	100	89	100

* Respondents did not answer every survey question, therefore, *n* = number of responses.

### Occupational therapists and teachers’ collaboration ratings

Data for the TTCI section of the survey were available for 81 occupational therapists and 27 teachers. Table 4 presents the total score on the TTCI and the scores for each individual component. Both teachers and therapists rated their collaborative practice positively (teachers *M* = 64.07, SD = 20.53, Mdn = 69 and therapists *M* = 73.25, *SD* = 16.85, Mdn = 74), and better than average (Mdn = 90). Professionals also rated their practice above average on each individual component of collaboration.

**Table 4.** Teacher-Therapist Collaboration Scores by component of collaboration and profession.

Component and range	Teachers (*n* = 27)			Occupational Therapists (*n* = 81)		
	Mean	SD	Mdn	Mean	SD	Mdn
Total TTCI (range 30–150, Mdn = 90)	64.07	20.53	69	73.25	16.85	74.00
Total interdependence (range 9–45, Mdn = 27)	18.11	6.34	18	20.88	5.75	20.5
Total newly created professional activities (range 4–20, Mdn = 12)	8.96	2.93	9	9.57	2.69	9
Total flexibility (range 5–25, Mdn = 15)	9.78	3.69	10	11.82	3.37	11
Total collective ownership of goals (range 5–25, Mdn = 15)	10.59	3.68	11	12.77	3.18	12
Total reflection on process (range 7–35, Mdn = 21)	16.63	5.62	17	18.41	4.88	18

* Respondents did not answer every survey question, therefore, *n* = number of responses.

### Comparison of occupational therapists and teachers’ collaboration ratings

Mann–Whitney *U* tests were used to explore significant differences in collaboration ratings between teachers and occupational therapists. Results revealed no significant difference in the way teachers and therapists rated their overall collaboration scores (teachers *n* = 27, Mdn = 69, therapists *n* = 81, Mdn = 74, *U* = 1340, *z* = 1.75, *p* = 0.08, *r* = 0.08).

To compare teacher ratings and therapist ratings for individual components of collaboration, Mann–Whitney *U* tests were employed. These revealed that teachers rated themselves more collaborative than occupational therapists on the components of flexibility (teachers *n* = 27, Mdn = 10, therapists *n* = 81, Mdn = 11, *U* = 1493, *z* = 2.61, *p* = .009, *r* = 0.25) and collective ownership of goals (teachers *n* = 27, Mdn = 11, therapists *n* = 81, Mdn = 12, *U* = 1410, *z* = 2.26, *p* = 0.024, *r* = 0.22). There were no statistically significant differences between teacher ratings and therapist ratings for all other individual components.

### Demographic and background variables and collaboration ratings

To explore whether collaboration ratings differed according to gender and employment hours, teacher and therapist total collaboration ratings were combined, and Mann–Whitney *U* tests employed. There were no statistically significant differences for ratings of collaboration according to gender (males *n* = 8, Mdn = 68, females *n* = 100, Mdn = 53, *U* = 291.5, *z* = –1.273, *p* = 0.203, *r* = 0.1) or employment hours (full-time *n* = 67, Mdn = 51, part-time *n* = 59, Mdn = 41, *U* = 1574.5, *z* = 1.273, *p* = 0.203, *r* = 0.1).

Kruskal–Wallis tests were conducted to explore the relationship between practitioners’ combined collaboration ratings and their state of practice, age group, level of education and number of years in the profession. They were also used to compare whether teachers and therapists’ ratings differed according to employer or model of therapy service. The results, presented in Table 5, revealed no significant differences.

**Table 5.** Results of Kruskal–Wallis Tests.

Variable	*n*	Degree of freedom	Chi square value	*p*-Value
Combined teacher-therapist TTCI score				
State of practice	108	2	1.16	0.56
Age group	109	4	2.77	0.60
Level of education	108	2	4.08	0.13
Number of years in profession	106	4	7.36	0.12
Teacher TTCI score				
Employer	27	2	2.35	0.30
Model of therapy service	27	3	4.57	0.20
Therapist TTCI score				
Employer	82	4	8.00	0.91
Model of therapy service	80	4	6.61	0.16

### Personal, professional and system influences and collaboration ratings

Personal, professional and systems influences were measured to understand the relationship between these and collaboration ratings. Table 6 reports the total influence ratings for both teachers (*n* = 26) and therapists (*n* = 79), as well as their ratings on the individual categories of influence. Both teachers and therapists’ ratings of total influences (Mdn = 57, teachers Mdn = 33.5, therapists Mdn = 38.00), as well as their ratings on individual categories, suggested these are acting as facilitators of collaboration. Notably, therapists’ ratings of systems’ influences suggests that these factors are verging on barriers, rather than facilitators (Mdn = 9, therapists Mdn = 9.00)

**Table 6.** Teacher-therapist influence Scores by influence category and profession.

Component and range	Teachers (*n* = 26)			Occupational therapists (*n* = 79)		
	Mean	SD	Mdn	Mean	SD	Mdn
Total influences score (range 19–95, Mdn = 57)	34.58	10.12	33.50	37.61	7.49	38.00
Personal influences score (range 8–40, Mdn = 24)	12.62	3.81	12.62	14.59	3.14	15.99
Professional influences score (range 8–40, Mdn = 24)	14.58	4.99	14.00	14.39	3.80	14.00
System influences score (range 3–15, Mdn = 9)	7.38	2.58	7.50	8.62	2.38	9.00

Results of Spearman’s rho tests, used to explore the relationship between combined teacher-therapist collaboration ratings and combined teacher-therapist influence ratings, demonstrated a large positive correlation (rho = 0.5–1) between the two variables (rho = 0.73, *n* = 106, *p* < 0.001), with greater supportive influences positively associated with greater levels of collaboration.

Spearman’s rho correlation tests were also used to explore the relationship between combined teacher-therapist collaboration ratings and each category of influence. Results indicate that professionals perceive all three factors to positively influence their collaboration ratings, as positive correlations were noted between collaboration ratings and personal factors (rho = 0.56, *n* = 106, *p* < 0.001), professional factors (rho = 0.63, *n* = 106, *p* < 0.001), and systems factors (rho = 0.67, *n* = 106, *p* < 0.001).

## Discussion

### Demographic and background characteristics

Collaboration literature reporting demographic and background data is scarce. Compared to available data the sample of occupational therapists and teachers in this study is similar to other studies, with some exceptions. This study reports that 24% of teachers held a master’s degree, and 73% a bachelors, compared to 55% and 40% in the study by Edick et al. (2022). The USA and Australia have different teacher education and licensing systems, which may explain this discrepancy.

Employment characteristics of occupational therapists differed between Australian studies. Kennedy and Stewart (2012) reported 11% of participants worked in private practice, and 39% worked full-time. The current study reported greater numbers of participants working in private practice (64%), and in full-time practice (55%). The study by Kennedy and Stewart (2012) was conducted in a different state, with a dissimilar history of government funded service integration in education (West et al., 2016). Their study was also conducted prior to the 2013 launch of the National Disability Insurance Scheme. This nationwide programme, which transformed disability services, led to a rise in private paediatric practitioners (Jackson et al., 2023). These factors may explain the differences between studies and have policy and practice implications.

### How do occupational therapists and teachers rate their own collaboration?

This study suggests that occupational therapists and teachers perceive themselves to be better than average collaborators. This both corroborates findings from previous studies (Friedman et al., 2022; Kennedy and Stewart, 2012; Rens and Joosten, 2014), and contradicts findings from other studies, that suggest ‘low’ levels of collaboration (Huang et al., 2011; Nochajski, 2002; Vlcek et al., 2020). These discrepancies may be explained in terms of methodology, population and context.

No previous studies measured collaborative practice using an instrument based on a conceptual framework and validated in schools with both teachers and therapists. As improvised measures lack rigour and standardisation, potentially leading to subjectivity and inconsistency in results, the use of non-standard measures prevents comparison and generalisation between studies. Friedman et al. (2022) did use a measurement instrument based on a conceptual framework; however, this was validated in the context of health (Archibald et al., 2014), not education, potentially impacting results.

Varied sample sizes and participants represent different study populations which may have influenced results. Although several studies recruited both occupational therapists and teachers (Friedman et al., 2022; Nochajski, 2002; Rens and Joosten, 2014; Vlcek et al., 2020), other studies recruited only teachers (Huang et al., 2011) or therapists (Kennedy and Stewart, 2012).

Finally contextual factors, such as geographic location and school culture, may explain the different findings between studies. Three studies were performed in the USA (Friedman et al., 2022; Huang et al., 2011; Nochajski, 2002). Although the rest were conducted in Australia, (Kennedy and Stewart, 2012; Rens and Joosten, 2014; Vlcek et al., 2020) they were in different states. Future research using instruments based on a conceptual framework with different populations could expand a common understanding of collaboration and provide more robust findings.

### How do occupational therapists and teachers’ collaboration ratings compare?

Total collaboration ratings did not differ significantly between teachers and occupational therapists; however, ratings on the components of flexibility and collective ownership of goals differed significantly. Teachers rated higher on both components.

Explanations may be postulated for why teachers rate themselves more collaborative than therapists in these components. Historically, students were removed from classrooms for therapy (Hutton, 2009). As therapy services are increasingly delivered in the classroom context (Koelbl et al., 2016; Missiuna et al., 2012), changes to teachers’ usual mode of working are necessary, as teachers accommodate therapists into what was traditionally their domain. This welcoming of therapists into their classrooms may be perceived by teachers as evidence of flexibility. As therapists are increasingly integrated into classrooms, teachers also find themselves incorporating non-academic goals into their work, despite their teaching ability being measured solely on students’ academic performance (Vlcek et al., 2020). This may be viewed as being flexible and more collaborative regarding goals. Finally, teachers may perceive flexibility in their interactions with therapists. As little to no formal time is given to teachers to collaborate with therapists (Wintle et al., 2017), meetings regularly occur in teachers personal time, lunch or recess breaks, or informally during teaching time (Barnes and Turner, 2001; Kennedy and Stewart, 2012). Teachers may perceive these accommodations as evidence of flexibility. As no other studies exist that directly compare collaboration scores of teachers and therapists this warrants further investigation with implications for professionals’ understandings on the role of collaboration in promoting inclusive education. Future research could expand to explore different practices utilised by professionals in their collaborative interactions.

### How do different variables correlate with collaboration ratings?

Various analyses demonstrated that teacher-therapist overall ratings were similar when compared for differences based on demographic and background variables. Combined ratings did not significantly differ by gender, age, education, employment, geographic location or model of service delivery. It is difficult to compare the results of this study with previous literature, as no studies exist that directly examine the relationship of these variables to measured collaborative practice.

The findings regarding years of experience in this study are contrary to Orentlicher et al. (2019), who reported that therapists with more than 10 years’ experience were more collaborative than those with less experience (Chi square = 9.95, *p* < 0.009, df = 3). Years of experience had no effect on collaboration ratings in this study (Chi square = 7.36, *p* = 0.12, df = 4). This incongruity may be explained by study population, location and measurement tool, as Orentlicher et al.’s (2019) USA study composed occupational therapists and physiotherapists and used a ‘non-standardised’ survey to measure collaboration. Conversely, Edick et al. (2022) also found years of experience mediated collaborative practice, noting that teachers with more than 10 years’ experience had less desire to learn from therapists.

That model of therapy service used by the occupational therapist had no effect on collaboration may refute the suggestion that different service models represent a continuum of collaboration (Rodger and Ziviani, 2006; Wintle et al., 2017). Although some view the direct pull-out model as least collaborative (Bayona et al., 2006) and the consultation model as most collaborative (Reid et al., 2006), the data support the notion that neither model is inherently more collaborative. Friend and Cook (2000) state that collaboration is a ‘style of interaction’ (p. 6) distinct from the activity in which it is occurring (Cook and Friend, 2010), thus, effective collaboration, the core of successful service provision (Anaby et al., 2019), is possible regardless of the model used.

Collaboration ratings were better than average and did not significantly differ based on demographic and background variables suggesting professionals perceive that they have the knowledge and skills to be successful in their collaborative endeavours regardless of age, gender, education, employment, location or service model. Findings suggest that collaboration is influenced by various factors. Future research could explore how these factors interact in specific contexts to identify ways to enhance collaborative practices.

### Personal, professional and system influences on collaboration

No previous studies have directly examined the relationship of personal, professional and systems factors to collaboration measures. This study suggests a positive relationship between each category of influence and collaboration ratings. Systems influences had a stronger relationship than personal or professional influences; however, the shorter subscale used to measure system influences may have influenced this relationship. That system influences have a stronger correlation than personal or professional factors corresponds to the literature, which consistently documents time as the biggest barrier to collaborative practice (Wintle et al., 2017). Unlike personal or professional factors, system factors are often outside an individual’s control. Improving collaboration may require changes to the systems that professionals work within, such as resourcing time off class for teachers to meet with therapists.

### Limitations and future research

The study is limited by its non-probability sampling technique, unknown response rate and small, unequal sample size. Limitations of the sampling technique relate to motivational bias and homogeneity. Participants are likely to respond because of strong feelings about the research, which they consider important. Similarly, as snowball sampling involves participants identifying other participants from the same population and supporting their access into the study, they are more likely to volunteer others who are similar to themselves (Saunders, 2012). Impediments related to the COVID-19 pandemic impacted recruitment, particularly teacher recruitment, resulting in a small, imbalanced sample.

While the expanded conceptual framework employed in this study provides a valuable foundation for understanding collaborative practice, the instrument’s reliance on self-reported data may introduce some bias. The TTCI measures perceptions of collaboration, rather than actual collaboration, and focuses on the process rather than the outcomes. As a key goal of collaboration is improving inclusion and student outcomes, this is an area of future research.

The study could be repeated with a larger sample of professionals from Australia, or from another country. The study could also be replicated with occupational therapist-teacher dyads to assess differences at a team level to identify areas of shared improvement.

## Conclusion and implications

The study pioneers the measurement of perceived collaborative practice between teachers and occupational therapists using an instrument grounded in a conceptual framework and validated within the school context by both professions. This ensures that what is being measured is truly collaboration (Ianni et al., 2022) and is the first step in determining the contribution of collaborative practice to successful inclusion.

Professionals could use the expanded version of Bronstein’s (2003) conceptual framework to support their understanding of collaboration and to understand the relationship and interaction between the components of collaboration. The framework provides a shared language and foundation for professionals to discuss their practice and may function as a practical tool that professionals could use to plan, execute and evaluate implementation efforts. The TTCI is a simple and quick tool which professionals could use individually, or jointly, to evaluate their collaborative practice and identify areas for improvement. Adequate measurement of collaborative practice allows professionals to evidence their collaborative endeavours, to recognise areas of success, and to identify areas for improvement. Using standardised measurement instruments aligned with a theoretical foundation provides a base for comparisons across different contexts and settings.

The study findings offer insights into the factors influencing collaborative practice in the context of inclusive education. As systems factors were a particular influence, findings may help to inform the development of strategies and policies aimed at promoting collaborative practice and improving inclusion.

**Key findings**

- Findings indicate that occupational therapists and teachers perceive their collaborations as effective; however, they could be further improved.
- A conceptual framework and associated instrument could be used by occupational therapists to plan, execute and evaluate their collaborative practice.
- Systems changes may be required to support improved collaborative practice.

**What the study adds**

- This study contributes evidence of the perceived effectiveness of collaborative practice between occupational therapists and teachers.
- Areas for improvement are highlighted and a practical framework and instrument to enhance collaboration is offered.
- The study emphasises the need for systemic changes to fully support collaborative practices.
